# Implementation and assessment of an end-to-end Open Science & Data Collaborations program

**DOI:** 10.12688/f1000research.110355.2

**Published:** 2022-12-05

**Authors:** Huajin Wang, Melanie Gainey, Patrick Campbell, Sarah Young, Katie Behrman

**Affiliations:** 1 University Libraries, Carnegie Mellon University, Pittsburgh, Pennsylvania, 15213, USA

**Keywords:** Open Science, Metascience, Academic Libraries, Program Assessment, User Data

## Abstract

As research becomes more interdisciplinary, fast-paced, data-intensive, and collaborative, there is an increasing need to share data and other research products in accordance with Open Science principles. In response to this need, we created an Open Science & Data Collaborations (OSDC) program at the Carnegie Mellon University Libraries that provides Open Science tools, training, collaboration opportunities, and community-building events to support Open Research and Open Science adoption. This program presents a unique end-to-end model for Open Science programs because it extends open science support beyond open repositories and open access publishing to the entire research lifecycle. We developed a logic model and a preliminary assessment metrics framework to evaluate the impact of the program activities based on existing data collected through event and workshop registrations and platform usage. The combination of these evaluation instruments has provided initial insight into our service productivity and impact. It will further help to answer more in-depth questions regarding the program impact, launch targeted surveys, and identify priority service areas and interesting Open Science projects.

## Introduction

The ways in which research is conducted are shifting toward more open, transparent and collaborative practices ( McKiernan *et al.*, 2016). This trend has been a response to changes in the funding and publishing landscape (e.g., open access mandates and open access publishing models; Davidson, 2005; Fyfe *et al.*, 2017; Kozlov, 2022), the nature of research collaboration (e.g., availability of digital collaboration platforms, trends in interdisciplinarity of research teams; Heller *et al.*, 2014; Cummings and Kiesler, 2014), the emergence of digital research infrastructures (e.g., open data repositories, open peer review platforms; Ponte et al., 2017) and cultural shifts in scientific practice (e.g., toward more open and transparent practices, open innovation; Huizingh, 2011). The term ‘Open Science’ has been used as an umbrella term to describe these trends. In 2018, Vicente-Saez and Martinez-Fuentes arrived at the following formal definition of Open Science through an analysis of ten years of scholarly literature on the topic: “[T] ransparent and accessible knowledge that is shared and developed through collaborative networks” ( Vicente-Saez and Martinez-Fuentes, 2018, p. 434). Similarly, Fecher and Friesike proposed five schools of thought that capture the breadth and complexity of the Open Science discourse, namely, schools focused on infrastructure, collaboration, public access to research, impact measurement and democratic principles ( Fecher and Friesike, 2014, p. 20). More recently, increasing reference has been made to UNESCO's definition of open science, which was defined as part of their recently adopted open science recommendations to inform global science policy-making. In this document, open science is defined as “an inclusive construct that combines various movements and practices aiming to make multilingual scientific knowledge openly available, accessible and reusable for everyone, to increase scientific collaborations and sharing of information for the benefits of science and society, and to open the processes of scientific knowledge creation, evaluation and communication to societal actors beyond the traditional scientific community.” ( UNESCO, 2021, p. 7).

Academic libraries played an early important role in the open science movement, particularly around open access publishing, which emerged in the 1990s with the development of scholarly publishing on the internet. The Scholarly Publishing and Academic Resources Coalition (SPARC) was formed by the Association of Research Libraries in 1997 to advocate for and promote open alternatives to the status quo of scholarly publishing, which was leading to rising cost burdens placed on libraries, researchers and academic institutions, and inequitable access to scientific knowledge ( Savenije, 2004). SPARC has since taken on issues of open data and open educational resources ( SPARC, 2022).

Another driving force in the open science movement has been the reproducibility crisis, which arose in psychological science in the mid 2000s and early 2010s ( Pashler & Wagenmakers, 2012). Questions arose at this time about the reproducibility of published research, thus calling into question the reliability of research findings, not just in psychology but across many scientific disciplines ( Ioannidis, 2005). Since this time, a variety of approaches have been developed to address this problem, such as pre-registration of research protocols, open peer review processes and journal requirements for data sharing. Many of these practices have been codified in the TOP (Transparency and Openness Promotion) Guidelines and have helped to further the open science movement ( Nosek *et al.*, 2015).

Continuing the shift towards more openness in research depends on many factors, including cultural and behavioral shifts amongst researchers, changes to incentive structures and publishing models, and infrastructural developments. While many research communities recognize the value of Open Science for furthering scientific knowledge, in actual practice, openness in research has been much more challenging to achieve ( Nosek *et al.*, 2015). Funders, publishers, and the public all play key roles in moving research toward open practices, as do institutions of higher education where incentive structures may run counter to a culture of research transparency. Despite this, there are various stakeholders in higher education settings that can foster Open Science practices. Moreover, Open Science overlaps with different areas of support across a university. For example, entities dealing with research integrity may take on the promotion of Open Science through a research transparency lens ( Bouter, 2018). Institutional research and analysis offices may have an interest in Open Science practices, as Open Science tools and platforms can assist with measuring and tracking research impact ( De Castro, 2018). Open Science initiatives may sprout from disciplinary or cross-disciplinary projects or sit within computer or data science departments. Examples of such initiatives include Stanford’s multi-school Center for Open and REproducible Science (CORES) and the Berkeley Initiative for Transparency in the Social Sciences (BITTS).

As Open Science has matured, academic libraries have leveraged the natural alignment to open science of existing services and principles related to information access and dissemination. For example, many libraries provide both infrastructure (e.g., institutional repositories) and funding (e.g., open access publishing funds) for sharing the products of research. Libraries also commonly provide training and support for managing research data, which relates to Open Science through the facilitation of practices that support data sharing and reuse. Recent literature suggests that libraries recognize their role in the Open Science movement, particularly in relation to repositories and open access publishing ( Ogungbeni *et al.*, 2018). Ayris and Ignat discussed important roles for libraries in Open Science in Europe ( Ayris and Ignat, 2018), and other research indicates a growing role for libraries in the Open Science landscape in Africa ( Siyao *et al.*, 2017; Tapfuma and Hoskins, 2019). Nonetheless, to our knowledge, the formalization of these tools and services in the form of “Open Science programs” in academic libraries is rare. Moreover, most libraries are likely not yet building programs with goals of providing a suite of tools and services to support Open Science throughout the research lifecycle.

**Figure 1.**
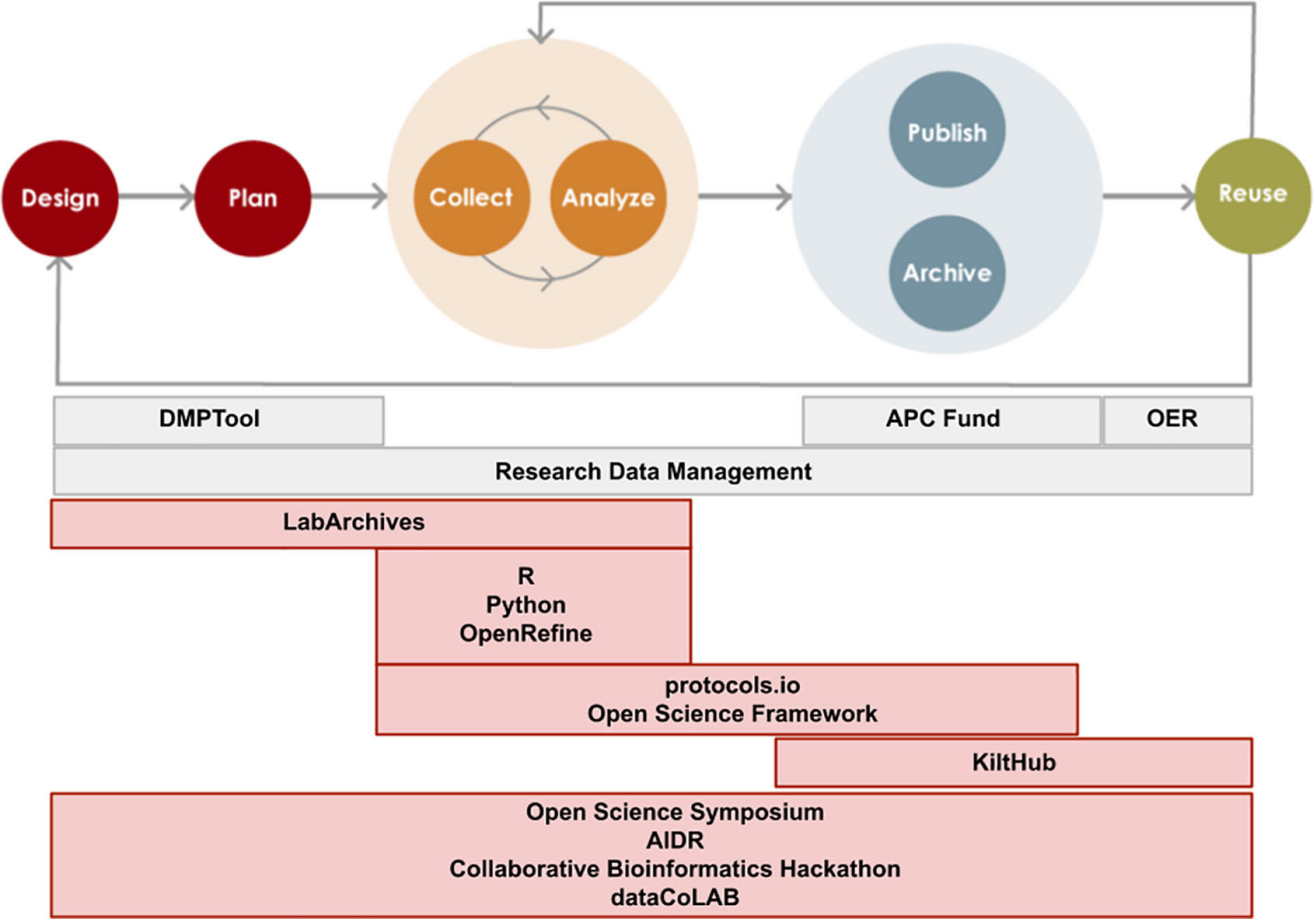
Open Science tools and services mapped to the research life cycle. Tools and services that OSDC supports with consultations, training opportunities, or licenses are mapped onto the phases of the research life cycle. Services and tools in gray boxes are supported by colleagues in the University Libraries that specialize in Open Access, Research Data Management, and Open Educational Resources. DMPTool: on online application that helps create Data Management Plans that meet funder mandates; OER: Open Educational Resources; APC: Article Processing Charge; OpenRefine: an open source digital tool for data cleaning and wrangling; KiltHub: CMU’s institutional repository; AIDR: Artificial Intelligence for Data Discovery and Reuse Conference; dataCoLAB: Data Collaborations Lab, an initiative to foster partnerships on data science projects on real-world research data.

Here, we present the framework for a novel Open Science program established in 2018 at Carnegie Mellon University (CMU) Libraries. The program, called Open Science and Data Collaborations (OSDC), encompasses a range of activities, tool support and training addressing Open Science practices throughout the research lifecycle ( Figure 1 and Table 1). Like other libraries, CMU Libraries also provide an institutional repository, a fund to partially cover author processing changes for open access publishing, and research data management services. While these services operate outside of the OSDC program umbrella, the programs and services work hand-in-hand to facilitate end-to-end Open Science practice, and much cross-team collaboration takes place. Therefore, we map these related services (gray boxes in Figure 1) together with those offered directly by OSDC to provide a bigger picture. The purpose of the current work is to present this model for a library-based Open Science program with a focus on program metrics and assessment. We begin with a brief environmental scan of Open Science activities at peer institutions. We follow with a logic model outlining our program activities, as well as short-, mid-, and long-term goals, and present examples of metrics that can be used and gathered to measure success. We conclude with a brief discussion of future implications for program planning and evaluation.

**Table 1.** Brief description of Open Science and Data Collaborations (OSDC) program components. Services in the OSDC program are composed of four major categories: tools, training, events, and collaboration.

Service	Description
**Tools**	
Open Science Framework (OSF)	Open Science Framework is an open-source web application for documenting and sharing project materials. OSDC provides an institutional license for OSF, as well as consultations and workshops to support use of it.
protocols.io	protocols.io is an open access repository for recording and sharing research methods and protocols. OSDC provides an institutional license for protocols.io, as well as consultations and workshops to support use of it.
LabArchives	LabArchives is a cloud-based Electronic Research Notebook (ERN) for documenting research. OSDC provides institutional licenses for the Education and Research editions of the platform, as well as consultations and workshops to support use of it.
KiltHub	Built on figshare and provided by CMU Libraries, KiltHub is CMU’s comprehensive institutional repository. It can be used to make any research product publicly available and citable. CMU Libraries provides data management and light curation support for researchers using the platform.
**Training**	
Carpentries Workshops	OSDC maintains a membership with the non-profit The Carpentries. We organize 2-3 day hands-on workshops on foundational computing and coding skills with Python, R, shell, Git, or OpenRefine with instructors and lesson plans from The Carpentries. Our membership also allows us to provide Carpentries Instructor training to a handful of researchers at CMU each year.
Libraries Workshop Series	Short workshops on open science tools and research practices, including short Carpentries-style workshops on R.
**Events**	
Collaborative Bioinformatics Hackathon	Hosted 1-2 times a year in partnership with other academic partners and DNAexus, the hackathon is a multi-day event that brings together academic and industry researchers from around the world to collaboratively work on crucial problems and opportunities in clinical bioinformatics. OSDC provides support on data management and sharing the outputs of the event.
Open Science Symposium	An annual symposium organized by OSDC that brings together researchers, funders, publishers, and tool developers to discuss the challenges and opportunities of Open Research.
AIDR (Artificial Intelligence for Data Discovery and Reuse)	An annual symposium organized by OSDC that focuses on harnessing the power of AI to accelerate the dissemination and reuse of scientific data and building a healthy data ecosystem.
**Collaboration opportunities**	
dataCoLab (Data Collaborations Lab)	Matches up researchers who want help with their datasets with consultants who have data science skills. Through weekly office hours and project-based consultations, this creates opportunities for people with different technical and disciplinary backgrounds to work together, following best practices that enhance reproducibility.

## Environmental scan

To evaluate the landscape of library Open Science programs, in the spring of 2021 we conducted an environmental scan of Carnegie Mellon University’s peer institutions, a list of 13 institutions of common qualities and goals (as defined by the Office of Institutional Research and Analysis at the university) ( Table 2). From the websites of each individual institution and each institution’s library, we searched for the general terms “open science,” “open scholarship,” and “open research” to attempt to locate similar programming and services to those offered by OSDC at CMU. We also searched for traditional Open Access resources, such as an institutional repository and an institutional Open Access policy to benchmark the number of peers with general Open Research services that may not be specifically described as “Open Science.” While the majority of peer institutions support open scholarship through open access policies and institutional and data repositories, dedicated open science centers and programming, either through the university library or through departmental structures, are less common ( Table 2).

**Table 2.** Summary of Open Science programs at Carnegie Mellon University (CMU)’s peer institutions. CMU’s peer institutions are California Institute of Technology, Cornell University, Duke University, Emory University, Georgia Institute of Technology, Massachusetts Institute of Technology, Northwestern University, Princeton University, Rensselaer Polytechnic Institute, Rice University, Stanford University, University of Pennsylvania, Washington University in St. Louis. The different levels of open science programming denoted in the table were defined as follows: Library Sponsored Open Science Programs: Full library-sponsored end-to-end Open Science programs similar to what CMU offers; Library Open Research Programming: Library-sponsored general open access/research/scholarship programs or units; Disciplinary Open Science Centers and Programs: Open Science programs and centers that are situated outside of or separate from the institution's library; Open Access Policies: Institutions with a policy or mandate for open access publishing; Institutional Repositories: Institutions with infrastructure for open sharing of research products and publications.

Library sponsored Open Science programs	Library Open Research programming	Disciplinary Open Science centers and programs	Open Access policies	Institutional repositories	Total peer institutions
0	4	5	10	12	13

In addition to manually checking the websites of peer institutions and to identifying any related programs outside of peer institutions, we ran a Google search using the following search string, which queried sets of search terms within three words of other search terms and limited the results to websites of U.S.-based postsecondary institutions: “open|reproducible|reproducibility AROUND(3) research|science|scholarship AROUND(3) institute|center|program” site:.edu. We then reviewed the results of the search until no relevant results were found on five consecutive results pages. No additional dedicated Open Science programs were identified with the Google search.

## Program implementation

In 2017, we began to develop services and initiatives to support open and reproducible research in response to the growing need for reliable infrastructure and training for Open Research practices ( Mckiernan *et al.*, 2016; Nosek *et al.*, 2015; AAU-APLU Public Access Working Group Report and Recommendations, 2017). What began as an *ad hoc* collection of services and collaborations was formalized as the Open Science & Data Collaborations (OSDC) Program in 2018. This program within Carnegie Mellon University Libraries consists of a team of subject librarians with deep research expertise and specialists in research data management and Open Data. While we have adopted the name “Open Science” due to its common use in the community, we support all types of research and often use the term “Open Research” to describe our activities. The OSDC program provides training and support for tools and practices that can be mapped onto the phases of the research life cycle ( Figure 1). Since our services together cover the entire life cycle, we describe the program as providing “end-to-end” support. The program has been in a phase of rapid expansion since its inception in 2018. We have leveraged our research experience, particularly in the life sciences, and our existing campus partnerships to develop new services that we believe will be of use and interest to the CMU community and help make research products open in accordance with the FAIR principles ( Wilkinson *et al.*, 2016).

Prior to the development of the OSDC program, CMU Libraries already provided extensive support for some areas of scholarship that are typically defined as Open Science, such as Open Access publishing ( Fecher and Friesike, 2014). Our comprehensive institutional repository, KiltHub, also predates the creation of OSDC. Currently, we collaborate with colleagues in the library that specialize in open access, research data management (RDM), and open educational resources (OER) to provide holistic support for open scholarship. These areas of Open Science that are outside of the purview of the OSDC program are not currently assessed by us ( Figure 1). In spite of the fact that KiltHub existed prior to the development of OSDC, we currently help support the platform and assess its usage as an integral piece of infrastructure for data sharing.

## Program assessment

As OSDC expands, one challenge has been getting structured and actionable feedback from the CMU research community, particularly from disciplines outside of the life and social sciences. To this end, we created a new arm of the program in 2021 that focuses on research and assessment. Our recent work has focused on developing a logic model and quantitative metrics on tool usage and event and training attendance. We will use this multi-pronged assessment approach to identify gaps in our service, shape the growth of the program in a data-driven and user-centered manner, identify future members for our Advisory Board, and create surveys designed for specific segments of our user community. Keeping the user in mind will be critical as the needs of the research community continue to evolve against the dynamic backdrop of data sharing mandates and the increasing desire for transparency and reproducibility in the research community.

### Logic model

**Figure 2.**
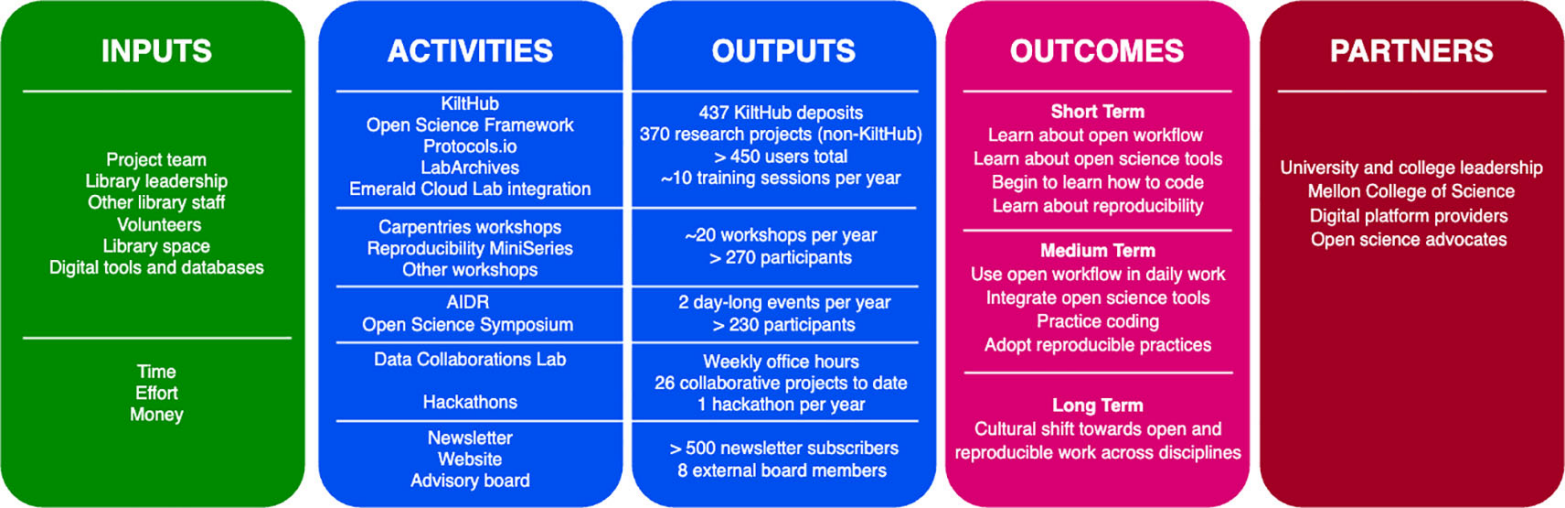
Graphic summary of a logic model. A logic model was created by listing inputs, activities, outputs, outcomes, and partners for each activity and creating a narrative. A simplified graphic summary was created to represent essential elements of the logic model. Inputs: resources required for all activities. Activities: the five groups of activities in the OSDC program; from top to bottom: tools, workshop, events, collaboration, and outreach. Outputs: product of each activity. Outcomes: short-, medium-, and long-term goals. Partners: partnerships formed to date. Emerald Cloud Lab: a remote controlled, automated lab where equipment is run remotely and workflow, data and code are automatically recorded; Reproducibility MiniSeries: short format workshop series that currently include R and OpenRefine.

The first component of our assessment strategy is a logic model ( Newcomer *et al.*, 2015) that provides a snapshot of the activities offered by the OSDC program and their respective outputs, resources needed to run the program, short-, medium-, and long-term goals to achieve for our users, and a list of partnerships formed through the program ( Figure 2). It provides a bird’s-eye view of the activities of the program and guides operational decisions and strategic planning. Values in outputs are estimated and serve as a baseline for further assessment. It should be noted however that values are not comparable between tools due to different time frames used for component datasets. The logic model will be reevaluated yearly.

### 5W1H metrics framework

To find more quantitative ways to measure program impact, we developed the second component of our current assessment strategy, a “5W1H” (Who, What, When, Where, Why, How) framework. Using this framework, originally developed for communication action research ( Yoshioka *et al.*, 2001), we developed metrics that use tool usage and event attendance data to help answer questions about our users and their use of our services.

First, we collected existing usage data across tool platforms. Specifically, we gathered usage data for the following tools: KiltHub, Open Science Framework, protocols.io, LabArchives. We also collected event registration data for Open Science-themed Libraries workshops, Carpentries workshops, Open Science Symposium, AIDR (Artificial Intelligence for Data Discovery and Reuse) Conference, and dataCoLAB (Data Collaborations Lab). We used event registration data as a proxy for event attendance since attendance data were not consistently collected. We expect, however, that registrations for events are higher than the actual attendance. Finally, engagement with the Open Science Newsletter, one of our core marketing tools, was also included in the assessment. The details of how data were collected for each of these services can be found in the Data Collection Methods section of this paper.

Data across platforms and events were cleaned and aggregated into a master dataset. The protocol we used to create the master dataset is published on protocols.io. We used Andrew IDs (CMU institutional emails) as unique identifiers for users of our services. Since the KiltHub dataset includes institutional and departmental affiliation data for all current CMU graduate students, staff, and faculty, we matched Andrew IDs for users of our other services to the KiltHub dataset. If users provided non-institutional email addresses, we queried their names in the CMU directory to determine their Andrew IDs, if possible. Undergraduates are represented in the dataset simply as “Undergraduates” since we could not consistently determine their departmental affiliation. We confirmed their status as undergraduates by querying their names in the CMU directory.

CMU and University of Pittsburgh (Pitt) have some joint centers and programs, and we noted that 60 users in the master dataset (5% of the total 1,348 unique users) have primary Pitt affiliations ( Table 3). For our analyses, we filtered out Pitt users. We also filtered out users from other non-CMU institutions or unidentified institutions (33%) and CMU users if we could not determine their departmental affiliation or if they were affiliated with administrative units on campus (4%). The total remaining records represented in the Results section (n=787) represents 58% of the total unique records (n=1,348) with which we began.

**Table 3.** Number and percentage of unique users by institution. In our analyses, we only included Carnegie Mellon University (CMU) users with known departamental affiliations (n=787). CMU users with unidentified or administrative affiliations (n=56), University of Pittsburgh users (n=60), or users at other or unidentified institutions were filtered out of the dataset (n=445).

Users	Count	Percent
Carnegie Mellon University (CMU)	787	58%
CMU with unidentified or administrative departments	56	4%
University of Pittsburgh (Pitt)	60	5%
Other or unidentified institution	445	33%
Total unique users	1348	100%

Based on the master dataset and platform-specific data, we generated a list of meaningful questions within the 5W1H framework ( Table 4). Metrics and their sub-variables were then defined to answer those questions. Currently, we are focusing on questions that we are able to answer readily with the data at hand, e.g.: who uses our tools and participates in our activities, which disciplines are the most engaged, and how do people use our tools and activities? Most of the metrics related to the questions require data collected from platform dashboards or provided by vendors. In other cases, the metric was derived from the dashboard or vendor data with simple calculations. For example, we can use data from the KiltHub dashboard to determine the institutional and departmental affiliation of each user. We can then derive the stage of the career of the user by querying institutional email addresses in the CMU directory. It should be noted that while we know that our users are largely in the Pittsburgh area, we do not collect any other information, such as IP addresses, that could be used to answer “Where” questions. Additional data collection is required to answer more nuanced questions about the impact and value of our services for users. Questions we eventually hope to address include: why do people use our tools or activities, how much value did we provide to users, and what impact are we making in people’s research process and in the whole research ecosystem?

**Table 4.** List of current metrics and associated variables. Metrics being used to evaluate the performance of the Open Science and Data Collaborations (OSDC) program and the variable(s) that are used to calculate each one. Metrics are organized using a “5W1H” (Who, What, When, Where, Why, How) framework representing the major classes of query the dataset is designed to answer. Data for each metric can either be collected directly from dashboards, vendors, or registration records, or derived from the direct data with simple calculations.

Question	Metric	Variable(s)	Source of data
**Who**	User affiliation	Institution, Department	Dashboard
	Stage of career	User type (faculty, postdoc, etc.)	Derived
	Superusers	Counts, Number of projects and registrations (all tools/events)	Derived
**What**	Number of users per tool	User (T/F) - all tools/events	Dashboard, vendor
	Number of tools/events used per user	User (T/F) - all tools/events	Derived
	Number of registrations per event	Count (all events/workshops)	Dashboard
	Number of attendances per event	Count (all events/workshops)	Dashboard
	Number of event/workshop registrations per user	Counts (all events/workshops)	Derived
	Departmental breakdown of users per tool/event	User (T/F), Institution, Department	Derived
	Career stage breakdown of users per tool/event	User (T/F), Career Stage	Derived
**When**	Growth rate (growth over time)	Number of users plus time/date field	Derived
	Activity over time	Output plus date/time fields	Derived
**Why**	User satisfaction * (qualitative and quantitative)	User comments/feedback	Advisory Board, surveys
	Financial metrics * (for users)	Cost savings	Vendors
**How**	Output (number of products, tasks completed, etc.)	Number of projects and registrations (OSF), number of notebooks (LabArchives), number of activities (LabArchives), number of protocols (protocols.io), count of events of each type attended (workshops, Carpentries, DataCoLAB, AIDR_OSS), Count_KiltHub (KiltHub)	Dashboard, vendors
	Reach	Open rate, Click rate (newsletter)	Dashboard

* Metric that we have partial data for and can be calculated in the future.

Importantly, the metrics can be applied to the program as a whole or to specific tools and services. The user affiliation metric will indicate whether we are achieving broad coverage of disciplines with the program. The superuser metric will help us identify Open Science advocates in our campus community that can support our outreach efforts and provide valuable feedback. We can also track adoption of specific services over time with our Growth Rate metric to examine trends in Open Science research and help guide decision making.

## Example applications of current metrics

Even though the framework is still a work in progress, limited by the state of existing data, it already allows us to ask simple questions. As a proof of concept, we provide a few examples of applying this framework to extract interesting patterns from existing data.

**Figure 3.**
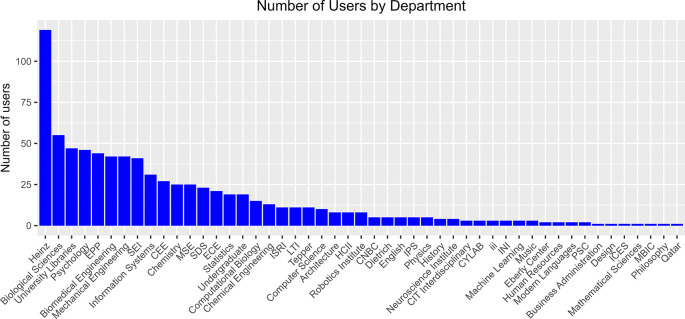
Departmental breakdown of all OSDC users. Number of users by department or academic unit, based on their primary affiliations. The main dataset integrating all usage data was used as input. Each user is counted only once even if they use multiple services. CNBC: Center for the Neural Basis of Cognition, CEE: Department of Civil and Environmental Engineering, CIT: College of Engineering, CYLAB: Security & Privacy Institute, ECE: Department of Electrical and Computer Engineering, EPP: Department of Engineering and Public Policy, MSE: Department of Materials Science and Engineering, Dietrich: Dietrich College of Humanities and Social Sciences, Heinz: Heinz College of Information Systems and Public Policy, HCII: Human Computer Interaction Institute, INI: Information Networking Institute, ICES: Institute for Complex Engineered Systems, IPS: Institute for Politics and Strategy, ISRI: Institute for Software Research, iii: Integrated Innovation Institute, LTI: Language Technologies Institute, MBIC: Molecular Biosensor and Imaging Center, PSC: Pittsburgh Supercomputing Center, SDS: Social and Decision Sciences, SEI: Software Engineering Institute, Tepper: Tepper School of Business.

To obtain an overview of disciplinary engagement, we summarized the number of users for each department, based on their primary affiliations ( Figure 3). The data came from the integrated dataset where usage of a given service or activity was represented as a “true/false” value. A user with a “true” in any of the services as counted as 1. These data show that the Heinz College of Information Systems and Public Policy has the highest number of users, followed by the Biological Sciences Department, University Libraries, and the Psychology department. We think this result can be partially explained by disciplinary culture as these disciplines are traditionally more engaged with library services and more active in the Open Science movement. Interestingly, some engineering and computer science departments also have high numbers of users, suggesting that we are starting to generate buy-in from these disciplines.

**Figure 4.**
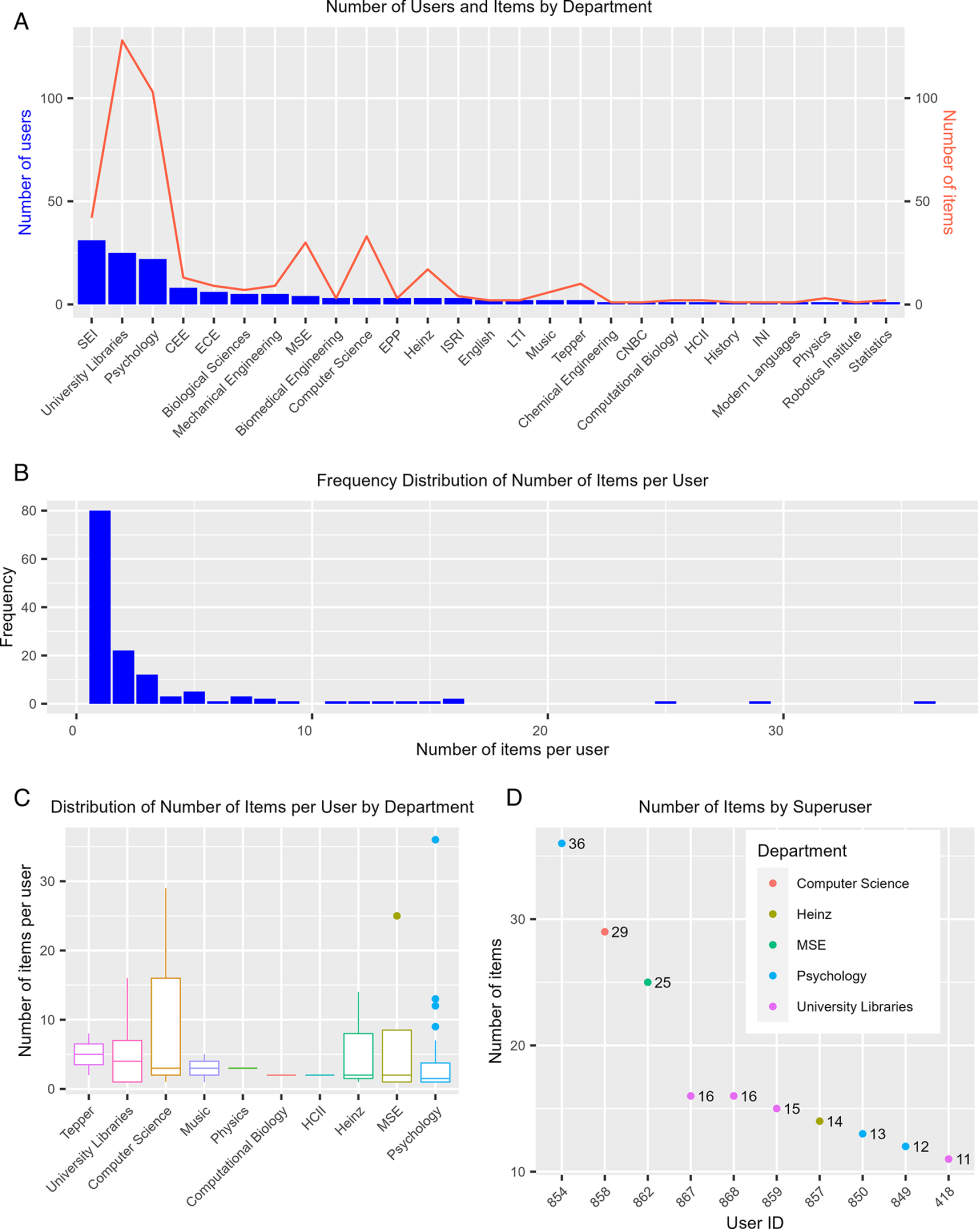
Summary of KiltHub use. (A) Departmental breakdown of number of KiltHub users (blue bars) and number of public items owned by users collectively in these departments (red line). (B) Frequency of number of public items owned per user (frequency is measured by the count of users with the specified number of public items owned). (C) Boxplot showing the distribution of the number of public items owned per user for the top 10 departments, measured by mean number of items per user. The boxed area represents the interquartile range (IQR), with the lower bar representing the first quartile (Q1) value, the intermediate bar representing the median value (Q2), and the top bar representing the third quartile (Q3) value. The lines, or “whiskers”, extending above and below the boxed area represent the range of values contained within 1.5 times the interquartile range (1.5 x IQR). Points extending beyond the whiskers represent outlier values (> 1.5 x IQR). (D) Number of public items owned for the 10 most active users (items > 10). These users are identified by their User ID (autonumber value assigned by Excel) to conceal the users’ identities. CNBC: Center for the Neural Basis of Cognition, CEE: Department of Civil and Environmental Engineering, ECE: Department of Electrical and Computer Engineering, MSE: Department of Materials Science and Engineering, Heinz: Heinz College of Information Systems and Public Policy, HCII: Human Computer Interaction Institute, INI: Information Networking Institute, ISRI: Institute for Software Research, LTI: Language Technologies Institute, SEI: Software Engineering Institute, Tepper: Tepper School of Business.

The number of users of each department does not necessarily reflect how active users from these departments are. Using KiltHub as an example, we further dissected the level of user activity for each individual tool hosted by the program. The reason we did not use the integrated master dataset for this purpose is that measurements between platforms, e.g., number of notebooks or number of registrations, are not comparable with each other. A breakdown of the number of users on KiltHub revealed that Software Engineering Institute (SEI), Psychology, and University Libraries, again, were among the departments or academic units that have the greatest number of KiltHub users ( Figure 4A, blue bars). However, when looking at user activity levels, specifically public items owned by users, those from SEI collectively owned fewer items compared to those from Psychology and University Libraries ( Figure 4A, red line). We further analyzed KiltHub usage at the level of individual users and saw different departments emerge when compared to the result from the total number of users. Among the top 10 departments ranked by the median values of the number of public items owned by each user in a given department, the School of Business ranked the highest, followed by University Libraries and the Computer Science Department ( Figure 4C). Even though the median values were relatively low overall – less than five items per user – some users owned much higher numbers of public items on KiltHub ( Figure 4C, outliers). This trend was also reflected at the level of the individual user, with the most active users owning more than 20 public items on KiltHub while the majority of users owned less than five items ( Figure 4B). We define users with more than 10 public items as “superusers.” We were able to identify these users (anonymized in this manuscript) and their department affiliations ( Figure 4D). The ability to identify superusers is especially valuable for collecting targeted feedback with interviews and surveys for service improvements in the future.

**Figure 5.**
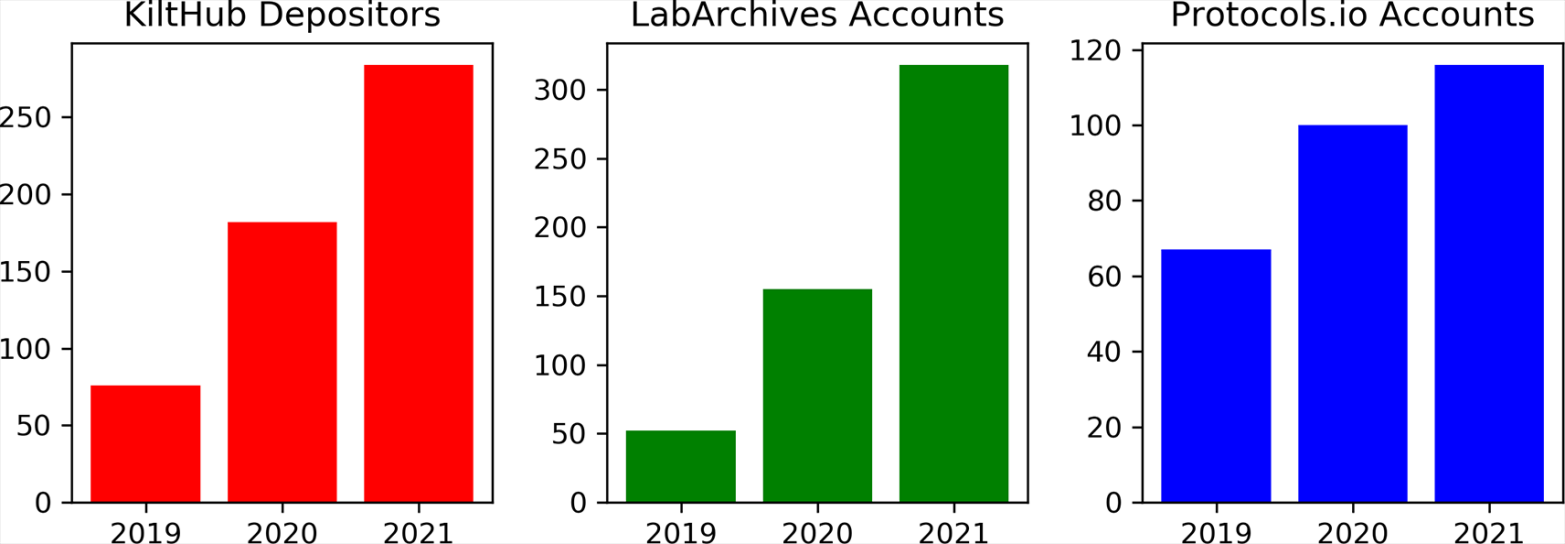
Tool usage over time (2019-2021). Number of total depositors on KiltHub, user accounts on LabArchives, and user accounts on protocols.io increased each year since the beginning of the program in 2018. Values presented are cumulative counts.

An important indicator of a program’s success is its growth over time. We used tool usage over time as a proxy to explore this question. By examining the total number of users who deposited data on KiltHub or the total number of accounts on LabArchives and protocols.io over time ( Figure 5), we found that there has been a steady increase in use every year since the inception of the OSDC program. This initial analysis establishes a useful baseline for future longitudinal studies.

## Discussion and future directions

Data sharing has represented a massive paradigm shift for research in recent years ( Gewin, 2016). This trend goes beyond data and applies to all research activities. In this paper, we use the term “data” loosely to refer to all research outputs including but not limited to data, code, and workflow. There are varying disciplinary norms and attitudes around data sharing and researchers often lack the training, time, infrastructure, or perceived incentives to openly share their research products. Fear that the data will be misused is another common concern ( Fecher *et al.*, 2015; Tedersoo *et al.*, 2021; Tenopir *et al.*, 2015). To address these challenges, we have created one of the first end-to-end Open Science programs sponsored by a library, with services that map onto all phases of the research lifecycle. One of our guiding priorities for creating Open Science services is that they have an impact on fostering collaboration and a cultural change towards research transparency. It is important, however, that we remain mindful of the barriers that researchers face. We therefore support a full gradient of Open Science practices, ranging from sharing research products publicly to improving the reproducibility of private workflows. For example, for researchers that are unable to share data due to working with sensitive data types, or are simply uncomfortable with data sharing, we might improve the reproducibility of their workflow for their future selves and collaborators. These types of consultations provide us with valuable opportunities to not only improve researcher experience around Open Science, but also discuss the benefits of publicly sharing research. Together with our community-building events, these types of interactions with researchers allow us to foster a culture of transparency.

As we continue to create services, we need to rely not only on conversations with researchers, but also on periodic quantitative assessments to understand their impact. The work presented here is the beginning of our program assessment and provides methods that we will update periodically and eventually supplement with additional metrics. This will allow us to focus our resources on priority areas, maximize the efforts of our small team, and guide our efforts to secure funding.

### Limitations of current data sources and future user data management strategy

Most of the data currently in our possession focuses on event registration and tool usage. Registration data is useful primarily for providing insights into, e.g., the popularity of specific OSDC initiatives (number of registrants, frequency of use, etc.), the reach/coverage across CMU and broader research community, specifically with regard to user type (student, faculty, etc.), institutional and departmental affiliation, and potential superusers. Our current data also include several variables related to the effectiveness of our various initiatives, e.g., number of items on KiltHub, number of projects and registrations on OSF, number of notebooks or activities on LabArchives, event attendance, or open and click rate of the Newsletter. However, we are only scratching the surface about the effectiveness or impact of the various OSDC initiatives; many deeper questions, e.g., how many publications, grant applications, career opportunities that we help users to obtain, and how much time we save users in their daily research, cannot be answered with the existing metrics. Developing metrics that reflect researchers’ productivity and success more directly would strengthen our value proposition to researchers and help them to rethink how productivity, efficiency, and impact are evaluated.

Despite these limitations, the current data and the 5W1H metrics framework will serve as a baseline to develop a strategy for user data management in the future to guide data collection, update, and analysis. A large part of our data collection process is limited by the platforms or tools that host the data. However, the usefulness of data can be improved by a few tweaks. To get the most out of our usage and registration data, a date field should be included for all relevant data tables, which will allow us to infer, for example, how the number of link clicks in a particular issue of our newsletter influences the number of registrations for specific events. Importantly, date information will help to control confounding factors when inferring whether the uptake in open science behavior is directly caused by the services we offer, or rather driven by important events and policy changes outside of the OSDC program. More generally, date information can reveal temporal patterns in the use of various tools/platforms and attendance at particular events, allowing us to better target our outreach efforts and workshops. Adding a date field will also allow us to track more meaningful changes in use after controlling for natural fluctuation patterns, which can in turn be used to estimate programmatic growth or decline.

To develop a more mature user data management system, metrics should be developed to provide insight into different stages of the research lifecycle ( Figure 1), particularly around the issues of productivity, efficiency, and impact. The specific variables that are relevant in each case will depend on the particular stage of the research lifecycle we are considering. For example, usage of protocols.io would likely reflect the data collection and analysis stage, while KiltHub usage more likely reflects the publishing and sharing stage.

We would also like to develop a more systematic data collection strategy that allows regular updates to data and results. The current data collection, cleaning, and analysis process is highly manual, making it time-intensive, error prone, and difficult to update. Developing an automated or semi-automated workflow would help to ease the administrative overhead on data updates and enable us to ask more longitudinal questions.

### Applications of the logic model and 5W1H framework

The combination of the logic model and the 5W1H framework provides complementary instruments to evaluate our program’s impact and to inform decision making. The logic model provides a bird’s-eye view of program activities and is an ideal tool for goal setting and communicating higher level ideas with leadership and stakeholders. The 5W1H framework, on the other hand, helps to evaluate and understand our activities and user engagement at a more granular level, making it possible to quantitatively assess our successes, identify areas for improvement, prioritize future work, and refine outreach strategies.

The most difficult thing in the metrics framework is the “why” question: what are the motivations for people to use our services? Is it to meet funder/publisher mandates, to get credit, or for other reasons? Developing such metrics would make it possible to quantitatively assess user motivation and productivity, evaluate the value and success of our services, and identify areas for improvement and prioritization in the future. For these types of questions, we would like to get direct feedback from users through surveys and interviews. To this end, the “superuser” metric ( Figure 4D) in the 5W1H framework helps to identify the right users to reach out to. We had initial success applying this metric to form a OSDC Advisory Board from our users, composed of graduate students, postdoctoral fellows, and faculty who are Open Science advocates and practitioners, and represent a variety of disciplines. The group meets 3-4 times a year to provide feedback in the style of a focus group on service updates, outreach strategies, and disciplinary practices and challenges.

Our work on the implementation of an end-to-end Open Science program and the development of assessment instruments will serve as a model that can be adopted by Open Science programs at other institutions, or other service-oriented organizations that wish to evaluate their success and impact. With further enrichment and adoption, the combined logic model and 5W1H framework we developed has the potential to grow into a benchmarking tool for equivalent programs and products that require both qualitative and quantitative assessment.

## Data collection methods

**KiltHub.** For the master dataset, profiles of all active users on or before 2 April 2021 were downloaded from the KiltHub Admin dashboard. We used the following data fields from the profiles for this study: ID, first and last name, email address, affiliation (department or center), and number of public items owned. Only data depositors with more than one public item owned were included in data analysis, while the names and email addresses of all users were used for data harmonization (see the published protocol for details). We filtered out private items since there are many reasons why a user might choose to keep their projects private. For usage over time, a separate dataset was downloaded from the dashboard that contains information about depositors.

**protocols.io**. Usage data including number of users, private, protocols, and public protocols were provided quarterly by the vendor and were collected for this study on 30 November 2021. Per protocols.io privacy policies, identifying information such as names, email addresses, or departmental affiliations were not shared. Therefore, these data were not included in the User Summary in Figure 3.

**Open Science Framework (OSF).** We collected user data from Open Science Framework (OSF) with our institutional OSF dashboard that includes first and last names and number of public projects and registrations on 19 January 2021. The number of public projects and registrations per user is the sum of these two metrics. Institutional emails were gathered by querying names in the Carnegie Mellon University Directory.

**LabArchives.** A Detailed Usage Report was downloaded from the Site Administrator dashboard. The report included first and last names, institutional email address, type of account (CE type), number of notebooks, and number of activities. For the purpose of this study, we were interested in Researcher and Instructor accounts. Student accounts were filtered out of the dataset. Data for the User Summary in Figure 3 were collected on 20 January 2021 and the usage over time data in Figure 4 were collected on 20 November 2021.

**Newsletter.** Newsletter data was accessed through Mailchimp. We were interested in users that routinely open the newsletter. To gather these data, we navigated to the Audience Dashboard and selected the Often segment under Engagement. This allowed us to collect data on our most engaged users, including first and last names and email addresses. We then searched user profiles in Mailchimp to gather data on Open Rate and Click Rate for each user. Newsletter data were collected on 20 January 2021.

**Events.** Event registration data for Open Science Symposium and AIDR were collected from the Indico, EasyChair, and EventBrite platforms. The data collected for each registrant included event name, first and last names, email address, and institution. Participation data from dataCoLAB were collected using a project intake form in Google Forms.

**Workshops and training.** Workshops and training related to Open Science at CMU are delivered primarily through two formats: (1) one- to two-hour workshops offered through the Libraries’ workshop series on the following topics: OpenRefine, Jupyter Notebooks, Open Science Framework, Data Management, and R, and (2) Carpentries workshops, which are two-to-three-day training sessions organized and managed by the Libraries’ Carpentries organizing team. Registration data were collected for Libraries’ and Carpentries workshops from LibCal and Eventbrite, respectively. Registration data, including first and last names and email addresses, were collected for each occurrence of a workshop that had occurred by 1 January 2021. All Libraries’ and Carpentries workshop data were combined for each workshop type (type defined by a combination of format and topic). Users were merged if they had used different emails for registration for different workshops, and it was clear from their name that they were the same person. For users that had registered for multiple occurrences of the same Libraries’ workshop, it was assumed that they had only attended one. For Carpentries workshops, we assumed that registrants may have attended more than one workshop even if it covered the same content. Libraries’ workshop data were then combined into a single dataset indicating whether or not a user had registered for a given workshop type and the total number of workshop types attended by each user.

### Ethical approval

After extensive communication with the Institutional Review Board (IRB), it was advised that as this project is intended for evaluation and improvement of internal processes without making generalizing statements, did not fall under the definition of research, and therefore did not require IRB approval. Informed consent for collecting the original data hosted by the university and the libraries was obtained by the university’s legal office. Data have been anonymized for this study before collection and analysis. Anonymizing the data does not change the scientific meaning of our findings.

## Data availability

Because original data used to develop assessment methods contain identifiable user information, they are only for internal use. Deidentified and aggregated data are openly available in KiltHub, CMU’s institutional repository (DOI: https://doi.org/10.1184/R1/19438586). Protocols used for data cleaning and processing are openly available on protocols.io ( https://doi.org/10.17504/protocols.io.b29gqh3w).
