# Supplementary material for: Implementation and assessment of an end-to-end Open Science & Data Collaborations program
Source: F1000Res. 2022 Dec 5;11:501. Originally published 2022 May 5. [Version 2] doi: 10.12688/f1000research.110355.2 (PMC9748376; doi:10.12688/f1000research.110355.2)
Supplement: Peer review history

# Peer review: Reviewer response for version 2

**DOI:** 10.5256/f1000research.140940.r157326

**Published:** 2022-12-13

**Authors:** Verena Heise

**Affiliations:** 1 Freelance Open Science Researcher, Gladbeck, Germany

I would like to thank the authors for taking the time to address my comments and provide a revised version of the manuscript. There are still two minor issues that I see. They do not necessarily need to be addressed in a second revised version of the manuscript but it would be good to record them here:

**1) The "end-to-end" model**

 In several places, the program is described as an end-to-end model for research projects and a slightly more cautious phrasing might be more appropriate. The program does offer very useful services across the stages of a research project but a) these services are not necessarily applicable to all types of research and only cover the "ends" of specific types of research, e.g. those that deal with mostly digital data, and b) an important stage of a research project is missing: the one where we develop hypotheses, e.g. through literature reviews.

**2) Figure 5**

 I still wonder how useful the data analysis shown in Figure 5 is. I see two problems here: a) the number of depositors/ accounts will always increase over time if you look at cumulative figures. A better way to assess growth would be to just identify the number of new depositors/ accounts per year. b) The number of accounts is not necessarily related to the number of “users”, i.e. just because someone has an account does not mean that they actively deposit information/ use a service. Maybe there is a better way to determine active tool usage, e.g. via the number of deposits per year, and one would then want to see whether the number of deposits increase per year.

Is the case presented with sufficient detail to be useful for teaching or other practitioners?

Yes

Is the work clearly and accurately presented and does it cite the current literature?

Yes

If applicable, is the statistical analysis and its interpretation appropriate?

Yes

Are all the source data underlying the results available to ensure full reproducibility?

Partly

Are the conclusions drawn adequately supported by the results?

Yes

Is the background of the case’s history and progression described in sufficient detail?

Partly

Reviewer Expertise:

Open Science, Reproducibility, Meta-research, Biomedical Research

I confirm that I have read this submission and believe that I have an appropriate level of expertise to confirm that it is of an acceptable scientific standard.

# Peer review: Reviewer response for version 2

**DOI:** 10.5256/f1000research.140940.r157325

**Published:** 2022-12-12

**Authors:** Guy A. Rouleau, Dylan Roskams-Edris

**Affiliations:** 1 Department of Human Genetics, The Neuro (Montreal Neurological Institute-Hospital), Department of Neurology and Neurosurgery, McGill University, Montreal, Quebec, Canada; 2 Tanenbaum Open Science Institute, The Neuro (Montreal Neurological Institute-Hospital), McGill University, Montreal, Quebec, Canada

We are happy with the revised version.

Is the case presented with sufficient detail to be useful for teaching or other practitioners?

Yes

Is the work clearly and accurately presented and does it cite the current literature?

Yes

If applicable, is the statistical analysis and its interpretation appropriate?

Yes

Are all the source data underlying the results available to ensure full reproducibility?

Partly

Are the conclusions drawn adequately supported by the results?

Yes

Is the background of the case’s history and progression described in sufficient detail?

Partly

Reviewer Expertise:

NA

We confirm that we have read this submission and believe that we have an appropriate level of expertise to confirm that it is of an acceptable scientific standard.

# Peer review: Reviewer response for version 1

**DOI:** 10.5256/f1000research.121952.r141852

**Published:** 2022-07-22

**Authors:** Verena Heise

**Affiliations:** 1 Freelance Open Science Researcher, Gladbeck, Germany

The article introduces an Open Science program developed at Carnegie Mellon University Libraries that supports researchers with tools, services and training to develop open and reproducible workflows. Additionally, it describes a framework for assessing the success of this program. Both of these aspects are very interesting for other institutions that are engaged in promoting open and reproducible research and would like to or are in the process of setting up similar services.

 The article is very informative but nevertheless I have a couple of major and minor comments that I hope will be helpful to the authors:

**Major items**

1. **The "end-to-end" model**
  In several places, the program is described as an end-to-end model for research projects and I wonder whether a slightly more cautious phrasing might be more appropriate. The program does offer very useful services across the stages of a research project but a) these services are not necessarily applicable to all types of research and only cover the "ends" of specific types of research, e.g. those that deal with mostly digital data, and b) an important stage of a research project is missing: the one where we develop hypotheses, e.g. through literature reviews. The latter might be part of the services offered but it is currently not mentioned in the manuscript.
2. **Citizen Science**
  This is mentioned in a couple of places (e.g. Figure 1) as a service supported by the OSDC (Open Science & Data Collaborations) but there is very little information on it in the paper. Could you clarify what the role of OSDC is with respect to Citizen Science? And just out of curiosity: Citizen Science can be included in research projects at all stages, so I was wondering why it only seems to be applicable to the data collection and analysis stage in Figure 1?
3. **Figure 1**
  I was wondering whether it might make sense to remove the grey boxes since the article mostly focuses on the OSDC. Two items that are not currently explained in the legend or the following table are the DMPTool and OpenRefine, so it would be great to add some more info on these.
4. **Table 2**
  It would be quite helpful to explain the categories in the table in a bit more detail. For example, what is the difference between Library sponsored OS programs and Library Open Research programming?
5. **Figure 2**
  Activities: I’m not sure what the Emerald Cloud Lab integration and Reproducibility MiniSeries refer to.
  Outputs: I guess the training sessions are related to the tools themselves? Would it make sense to have these in the second category together with the other workshops?
  Weekly office hours: I was wondering why this is relevant. I guess the question would be how many people actually come to get support during the office hours?
  Outcomes (this is a conceptual point, so does not necessarily need to be addressed for this paper): I’m surprised they are defined as outcomes for users, not the OSDC. This might just be the way the strategic goals are defined for OSDC and it’s absolutely fine if the figure reflects these. I wonder whether that’s the best way to define outcomes for the OSDC because it's probably difficult to assess whether the OSDC has been successful with that selection of outcomes, especially the medium and long-term ones. For example, shifts in research culture require much more than just availability of training, tools and services, so it's not only down to the OSDC whether those cultural shifts actually materialise.
6. **Figure 3**
  As far as I can see the acronyms EPP, CIT, CYLAB are not covered in the figure legend. And another conceptual comment that does not necessarily need to be addressed here: it might be interesting to look at user numbers in relation to size of the institutes, e.g. percentage of faculty using services, so that institutes of different sizes can be compared quite easily.
7. **Figure 5**
  This is a very stupid question but it would be great to clarify that these are new items/ new registrations per year and not the total numbers (e.g. of accounts) in that year, which would be the sum of accounts from the previous year plus new accounts?

**Minor items**

**Introduction**

1. “This trend has been a response to changes in the funding and publishing landscape, the nature of research collaboration, the emergence of digital research infrastructures and cultural shifts in scientific practice” - I wonder whether a couple of references might help here or a some examples of the changes this refers to. I guess it refers to changes such as open access/ open data mandates but it would be helpful to see what the authors mean.
2. Definition of Open Science: I wonder whether it might be helpful to include the definition used in the UNESCO recommendation (https://en.unesco.org/science-sustainable-future/open-science/recommendation) because it's a definition of Open Science that is gaining quite a bit of traction, especially among policy makers.

**Discussion**

1. I was wondering why the first paragraph is mostly about data sharing and does not cover Open Science practices more broadly since OSDC is about more than just data sharing.
2. The sentence that ends with “prevents us from being able to make a clear value proposition to researchers for whom productivity, efficiency, and impact are the most important factors” might need rephrasing. I would hope that the impact of reproducible workflows and data management are obvious to many researchers because they have clear impacts on efficiency and impact (e.g. see higher citation rates of openly available papers and data). I understand that it can be difficult to quantify some of those aspects but nevertheless there is a clear value proposition for using open/ reproducible workflows and a number of papers have dealt with the selfish reasons for working in a more open/ reproducible way (e.g. McKiernan paper cited already and https://genomebiology.biomedcentral.com/articles/10.1186/s13059-015-0850-7)
3. There are a couple of sentences about data management, e.g. this one: *"the current data and the 5W1H metrics framework will serve as a baseline to develop a strategy for data management in the future to guide data collection, update, and analysis”*. I think this refers to management of user data related to services and not research data itself and it would be great if that could be rephrased slightly to make the distinction more obvious.

Is the case presented with sufficient detail to be useful for teaching or other practitioners?

Yes

Is the work clearly and accurately presented and does it cite the current literature?

Yes

If applicable, is the statistical analysis and its interpretation appropriate?

Yes

Are all the source data underlying the results available to ensure full reproducibility?

Partly

Are the conclusions drawn adequately supported by the results?

Yes

Is the background of the case’s history and progression described in sufficient detail?

Partly

Reviewer Expertise:

Open Science, Reproducibility, Meta-research, Biomedical Research

I confirm that I have read this submission and believe that I have an appropriate level of expertise to confirm that it is of an acceptable scientific standard, however I have significant reservations, as outlined above.

## References

[1] : Five selfish reasons to work reproducibly. *Genome Biol* .2015;16: 10.1186/s13059-015-0850-7 274 10.1186/s13059-015-0850-7 26646147PMC4673789

# Peer review: Reviewer response for version 1

**DOI:** 10.5256/f1000research.121952.r137000

**Published:** 2022-07-19

**Authors:** Guy A. Rouleau, Dylan Roskams-Edris

**Affiliations:** 1 Department of Human Genetics, The Neuro (Montreal Neurological Institute-Hospital), Department of Neurology and Neurosurgery, McGill University, Montreal, Quebec, Canada; 2 Tanenbaum Open Science Institute, The Neuro (Montreal Neurological Institute-Hospital), McGill University, Montreal, Quebec, Canada

This article is about the development of a program created by the Carnegie Mellon University Libraries to support Open Science practice at CMU, in particular by offering educational and support services around various platforms.

 A good paper describing an important initiative. Their development of a unified program, as well as the generation of useful metrics, are an important contribution to the development of Open Science at institutions.

 As discussed in the comments below, there is some information they could add about the history of open science/open research that would add clarity to the text, as well as a few minor revisions that should be made to supply necessary information in figures.

**1. Greater discussion of the historical role of libraries in open research practices.**

 In the introduction the authors use the sentence *“As Open Science has matured, academic libraries have also entered this space leveraging the natural alignment with existing services and principles related to information access and dissemination.”* This sentence somewhat mischaracterizes the historical role of libraries in the Open Science movement. To a significant extent open science emerged from the Open Access movement, and libraries have been heavily involved in that movement since at least 1997 when the Association of Research Libraries founded Scholarly Publishing and Academic Resources Coalition (SPARC). It would be more accurate to say that the role of libraries has always been important to the movement to open science, but that they have more recently started to play an instrumental role beyond open access to publications.

**2. Earlier introduction to the relationship between reproducibility and open science.**

 The authors introduce the concept of reproducibility on page 5, both in the google search string they used to quality control their search of peer institutions and in the first sentence in the section on “Program Implementation.” To readers versed in Open Science this connection will likely come as no surprise, but to the less versed reader the reference to reproducibility comes somewhat out of the blue. Why, for example, should that be included in a search for Open Science relevant programs? A brief explanation, perhaps in the introduction near where they address the “five schools of thought” would make their search strategy and the other references to reproducibility clearer. This observation is also relevant to the comment below on the history of the field of psychology and its relevance to the development of Open Science.

**3. Further historical context around the field of psychology.**

 Similar to the comment on the history of libraries, and related to the comment on reproducibility, there are several ways a brief discussion of the history of the relationship between psychology and open science would add clarity to the paper. Unlike with libraries’ advocacy for Open Access, which fed into Open Science and which were primarily concerned with rising subscription fees and the efficient dissemination of knowledge, psychology researchers have been a major force in the history of Open Science primarily as an aid to reproducibility and replicability.

 Adding some content about this history would help both to make clear the relationship between reproducibility/replicability and open science—indeed psychologists were the first to identify it as a crisis ( https://journals.sagepub.com/doi/10.1177/1745691612465253) – but would also contribute to why the CMU Psychology department has such a large number of users (see Fig. 3).

**4. Adding some discussion of the difficulties in sharing sensitive human data and what this might imply for the data collected.**

 While the authors address the difficulties posed by ethical constrains on sharing sensitive human data in the section “Discussion and future directions”, acknowledging this particular difficulty in earlier sections where they discuss the data around departmental users would add important interpretive context for why departments that deal with this kind of data regularly (for example the Centre for the Neural Basis of Cognition) might show fewer users.

**5. Acronyms used in Figure 3.**

 The “EPP” acronym on the x-axis in Figure 3 is not explained in the text below the figure, though it likely refers to the department of Engineering and Public Policy. Also, the use of “iii” rather than “III” for the Integrated Innovation Institute isn’t consistent with the capitalization of the other acronyms, though there may be a reason for this I am not aware of.

**6. Note on important policy changes that may impact metrics**

 While it may not be possible in the context of this paper, it would be useful in future work for the authors to develop a way of assessing important events at the institutional, state, and national levels that impact the uptake of Open Science practices. The emergence of, for example, the NIH policy on data management and sharing may have a significant impact on their metrics in the absence of activity by the OSDC itself. They do address this to some extent in the proposed discussions with “superusers” in the section “Applications of the logic model and 5W1H framework”, and it will be very difficult to give a fully causal account, but at least acknowledging this confound further, or creating some automated system through google alerts or scraping relevant twitter hashtags/keywords (e.g., “Open Science” + “Policy” + filtering for CMU, state, and/or national users) could help provide such a timeline. If, for example, they see an increase in some metrics in the absence of events in the timeline, they can be more certain that the effect is endogenous.

Is the case presented with sufficient detail to be useful for teaching or other practitioners?

Yes

Is the work clearly and accurately presented and does it cite the current literature?

Yes

If applicable, is the statistical analysis and its interpretation appropriate?

Yes

Are all the source data underlying the results available to ensure full reproducibility?

Partly

Are the conclusions drawn adequately supported by the results?

Yes

Is the background of the case’s history and progression described in sufficient detail?

Partly

Reviewer Expertise:

Neuroscience, Genetics, Neurology, Open Science

We confirm that we have read this submission and believe that we have an appropriate level of expertise to confirm that it is of an acceptable scientific standard, however we have significant reservations, as outlined above.

## References

[1] : Editors' Introduction to the Special Section on Replicability in Psychological Science: A Crisis of Confidence?. *Perspect Psychol Sci* .2012;7(6) : 10.1177/1745691612465253 528-30 10.1177/1745691612465253 26168108
